# Characterization and treatment of landfill leachate membrane concentrate by Fe^2+^/NaClO combined with advanced oxidation processes

**DOI:** 10.1038/s41598-018-30917-5

**Published:** 2018-08-21

**Authors:** Meng Qiao, Xu Zhao, Xiaoyun Wei

**Affiliations:** Key Laboratory of Drinking Water Science and Technology, Research Center for Eco-Environmental Sciences, Chinese Academy of Sciences, Beijing, 100085 China

## Abstract

Landfill leachate membrane concentrate (LLMC) is a type of non-biodegradable wastewater intercepted by the membrane filtration of the landfill leachate membrane bioreactor (MBR) effluent. The concentrations of chemical oxygen demand (COD) and ammonia nitrogen (NH_4_^+^-N) in the LLMC collected from a landfill in Beijing were determined to be 4700 mg/L and 487 mg/L, 2–5 times higher than those in the MBR effluent. The photoelectro oxidation (PEO) followed with the NaClO enhanced Fe^2+^ coagulation were more effective for the removal of COD than the Fenton oxidation followed with the enhanced coagulation. The final removal efficiencies of COD, UV_254_, NH_4_^+^-N and color degree were 86%, 95%, 93% and 95% with Fe^2+^ (90 mmol/L) and NaClO (60 mmol/L, Fe^2+^:NaClO = 1.5:1), and PEO for 3 hours with a current density of 400 A/m^2^. Due to the existence of Cl^−^, the chlorinated intermediates, which would be more toxic, were detected in the PEO treatment. However, the intermediates could be eliminated finally. As a result, the NaClO enhanced Fe^2+^ coagulation treatment combined with PEO treatment was efficient for the treatment of LLMC.

## Introduction

Landfill leachate is generated from sanitary landfills, including the inherent water in the sanitary landfill, the water generated from the biological process, the underground water permeated in the landfill, and the rainy and snow water enter the landfill as the major source. Landfill leachate contains persistent organic components^1,2^. Landfill leachate is usually treated by biological treatment firstly. Membrane bioreactor (MBR) is widely used in the treatment of the landfill leachate^3,4^. The Pollution Control Standard for Domestic Waste Landfill has become stricter in China since 2008. Thus, the membrane treatment, including nanofiltration or reverse osmosis membrane has been used in the further treatment of the effluent of MBR (EMBR)^5–7^. The problem was the generation of the concentrated leachate incepted by the membrane (landfill leachate membrane concentrate, LLMC) in the membrane treatment. The organic contaminants, ammonia nitrogen (NH_4_^+^-N), heavy metal and salinity, were concentrated compared with the EMBR. The dissolved organic matters (DOM) in the LLMC could absorb micro organic pollutants, as well as complex heavy metals. As a result, the humic acid (HA), fulvic acid (FA) fractions in the DOM were toxic to organisms, confirmed by luminescent bacteria^8^. Recently, the LLMC was usually recirculation to the bioreactor or landfills^5^. If the concentrate leachate was recirculated, the concentration of chemical oxygen demand (COD) and ammonia nitrogen (NH_4_^+^-N) would be increased by 29% in the first 5 years and by 53% in 10 years^9^. If the LLMC underwent biological treatment, NH_4_^+^-N might be inhibit the activity of the microorganisms^10^. Therefore, it is imperative to develop an effective way for the treatment of the LLMC, especially the organic contaminants and NH_4_^+^-N.

Generally speaking, the biodegradability of the LLMC was poor^11^. Therefore, the biological treatment might be ineffective for the elimination of the organic matters in the LLMC. Coagulation was a commonly used technology for the elimination of the non-biodegradable organic matters^12^. Particularly, the ferric salts coagulation was more effective than the aluminum salts for the elimination of the organic compounds^13^. Ma *et al*. pointed out that the *in-situ* formation of ferric ion was more efficient for the coagulation of organic compounds than the direct addition of ferric salts^14^. NaClO was usually used for the oxidation of NH_4_^+^-N^15,16^. Meanwhile, NaClO could enhance the *in-situ* generation of ferric ion from Fe^2+^. NaClO enhanced Fe^2+^ coagulation has been firstly used for the treatment of the EMBR^12^. The removal efficiencies of COD in the EMBR increased from 24% with Fe^2+^ coagulation treatment to 54% with NaClO enhanced Fe^2+^ coagulation^12^. Therefore, it was supposed that NaClO enhanced Fe^2+^ coagulation would also be efficient for the treatment of LLMC with higher concentration of both COD and NH_4_^+^-N. In order to further increase the removal efficiency of COD and NH_4_^+^-N, advanced oxidation processes (AOPs) were needed. AOPs were attractive methods for the elimination of non-biodegradable organics, as well as increase the biodegradability of the LLMC^5^. After 110 min continuously ozone generation-reaction treatment, COD and NH_4_^+^-N could be removed 56% and 0%, respectively^5^. During the Fenton oxidation, the highest removal efficiency of COD in the LLMC was 60%^8^. While with the treatment of a two-stage enhanced coagulation followed with photoelectro oxidation (PEO), 86% of COD in the LLMC could be removed^17^. So we proposed that the combination of the coagulation and PEO might significantly improve the removal efficiency of COD.

Herein, the objective of this study was to seek for a suitable technique for treatment of the organic compounds and NH_4_^+^-N in the LLMC. A typical LLMC collected from a landfill in Beijing was characterized. NaClO enhanced Fe^2+^ coagulation combined with two kinds of AOPs, including Fenton oxidation and PEO, were used for the treatment of LLMC. Specifically, the chlorinated intermediates were identified in the PEO and electro oxidation (EO) treatment processes.

## Results and Discussion

### Characterization comparison of the LLMC with the EMBR

The regular chemical parameters of LLMC and the EMBR are listed in Table 1.

**Table 1:** Chemical properties of landfill leachate membrane concentrate (LLMC) and effluent of MBR treated landfill leachate (EMBR).

	LLMC (BJ)	EMBR (BJ)	LLMC (ZJ-X)	LLMC (ZJ-S)	LLMC (ZJ-N)	LLMC (QD-RO)	LLMC (QD-NF)
COD (mg/L)	4700 ± 121	1200 ± 22	1158	400	1165	2356	5846
NH_4_^+^-N (mg/L)	487 ± 9	90 ± 3	46	78	298	65	43
pH	8.0 ± 0.03	6.3 ± 0.02	5.6	7.6	5.6	7.8	7.3
Cl^−^ (mg/L)	10458 ± 205	4960 ± 64	1779	1000	3999		
Color degree (°)	3.20 ± 0.08	0.76 ± 0.03					
Turbidity (NTU)	3.62 ± 0.10	1.24 ± 0.03					

BJ-Beijing; ZJ-Zhejiang; QD-Qingdao; X, S, N-different landfill sites in Zhejiang; RO-reverse osmosis; NF-nanofiltration.

The average concentrations of COD and NH_4_^+^-N in the LLMC were 4700 mg/L and 487 mg/L respectively, about 2–5 times higher than those in the EMBR (1200 mg/L and 90 mg/L). Compared with the LLMC in Zhejiang province and Qingdao, the concentrations of COD and NH_4_^+^-N in Beijing were much higher^5,11^. The LLMC was weakly alkaline (pH = 8.0), while the EMBR was weakly acidic (pH = 6.3). The concentrations of Cl^−^ (10458 mg/L) were also much higher than those in Zhejiang (1000–3999 mg/L).

**Figure 1:**
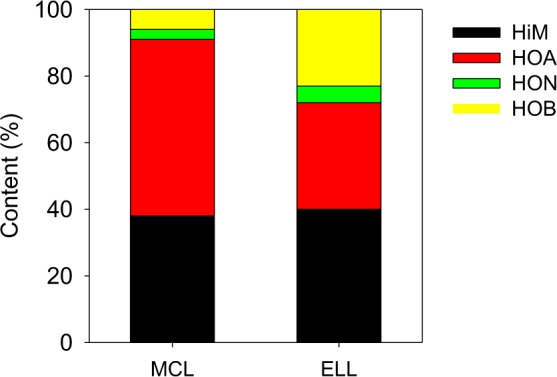
Hydrophilic and hydrophobic fractions in the LLMC and EMBR characterized with UV_254_ (HiM-hydrophilic matter, HOA-hydrophobic acids, HON-hydrophobic neutrals and HOB-hydrophobic bases).

The DOM in the LLMC and EMBR were fractioned by resin (Fig. 1). The DOM of both LLMC and EMBR, characterized with UV_254_, were mainly composed with hydrophilic matter (HiM) and hydrophobic acids (HOA). Compared with the EMBR, the LLMC contained more HOA and less hydrophobic neutrals (HON) and hydrophobic bases (HOB). The hydrophobic fractions were easier eliminated than the hydrophilic fractions using the chemical treatment^12^.

The ratios of the absorbance A_253_/A_203_ reflected the types of the aromatic ring substituent groups, including the aliphatic chain with lower A_253_/A_203_ values, the carbonyl, carboxyl, hydroxyl and esters with higher A_253_/A_203_ values^18^. It was reported that the A_253_/A_203_ ratios equaled to 0.02, 0.21 and 0.42 were estimated for aliphatic, hydroxyl and carboxylic substitutions of DOM, respectively^19^. Compared different fractions in LLMC and EMBR, HOA > HOB > HON > HiM, indicating that the substituent groups in the HiM fraction was mainly aliphatic chain, and the content of carbonyl, carboxyl, hydroxyl and esters were higher in the HOA fraction (Table 2). Similar result was obtained by a previous study^20^. The LLMC contained more carbonyl, carboxyl, hydroxyl and esters than the EMBR.

**Table 2:** A_253_/A_203_ in different element in the landfill leachate membrane concentrate (LLMC) and effluent of MBR treated landfill leachate (EMBR).

	HOA	HON	HOB	HiM
LLMC	0.232	0.104	0.158	0.045
EMBR	0.081	0.042	0.064	0.025

HOA-hydrophobic acids; HON-hydrophobic neutrals; HOB-hydrophobic bases; HiM-hydrophilic matter.

### Treatment of the LLMC using NaClO enhanced Fe^2+^ coagulation

**Figure 2:**
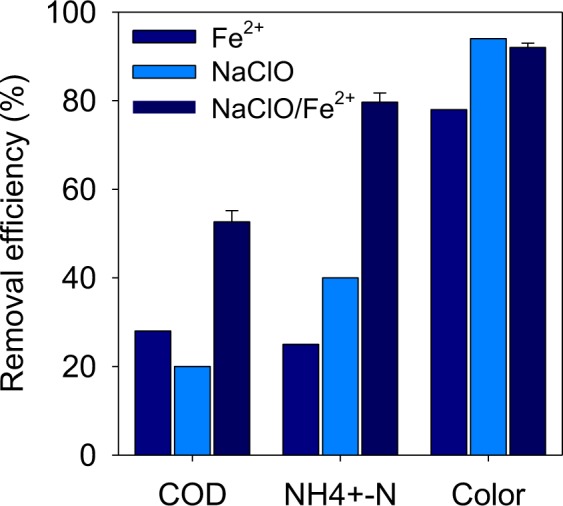
Final removal efficiencies of COD, NH_4_^+^-N and color degree by NaClO enhanced Fe^2+^ coagulation and individual Fe^2+^, NaClO coagulation (Fe^2+^: 90 mmol/L, NaClO: 60 mmol/L).

NaClO enhanced Fe^2+^ coagulation effectively improved the removal efficiencies of COD and NH_4_^+^-N (53% and 80%) compared with individual Fe^2+^ coagulation (28% and 25%) or NaClO oxidation (20% and 40%) (Fig. 2). Fe^2+^ could eliminate organic compounds through coagulation. NaClO could oxidize organic compounds into small molecular weight matters, CO_2_ and H_2_O. Additionally, the formation of ferric hydroxide from Fe^2+^ could be enhanced by NaClO besides the dissolved oxygen in water Eq. (1)^12^. Thus, the removal of organic compounds and NH_4_^+^-N could be enhanced through coagulation. From the aspect of ammonia, NH_4_^+^-N could be oxidized by sufficient HClO into N_2_ finally Eqs (2–6)^12^. The removal mechanism was similar to the “Breakpoint Reaction”^21^. Therefore, the removal efficiencies of COD and NH_4_^+^-N in the NaClO enhanced Fe^2+^ coagulation treatment were 1–2 times higher than the individual Fe^2+^ coagulation or NaClO oxidation treatment.

1 $${\rm{N}}{\rm{a}}{\rm{C}}{\rm{l}}{\rm{O}}+2{\rm{F}}{{\rm{e}}}^{2+}+4{\rm{O}}{{\rm{H}}}^{-}+{{\rm{H}}}_{2}{\rm{O}}\to 2\mathrm{Fe}{({\rm{O}}{\rm{H}})}_{3}+{\rm{NaCl}}$$

2 $${\rm{NaClO}}+{{\rm{H}}}_{2}{\rm{O}}\to {\rm{HClO}}+{\rm{N}}{\rm{a}}{\rm{O}}{\rm{H}}$$

3 $${\rm{H}}{\rm{O}}{\rm{C}}{\rm{l}}+{\rm{N}}{{{\rm{H}}}_{4}}^{+}\to {\rm{N}}{{\rm{H}}}_{2}{\rm{C}}{\rm{l}}+{{\rm{H}}}_{2}{\rm{O}}+{{\rm{H}}}^{+}$$

4 $${\rm{H}}{\rm{O}}{\rm{C}}{\rm{l}}+{\rm{N}}{{\rm{H}}}_{2}{\rm{C}}{\rm{l}}\to {\rm{N}}{\rm{H}}{\rm{C}}{{\rm{l}}}_{2}+{{\rm{H}}}_{2}{\rm{O}}$$

5 $${\rm{H}}{\rm{O}}{\rm{C}}{\rm{l}}+{\rm{N}}{\rm{H}}{\rm{C}}{{\rm{l}}}_{2}\to {\rm{N}}{\rm{C}}{{\rm{l}}}_{3}+{{\rm{H}}}_{2}{\rm{O}}$$

6 $$2{\rm{N}}{{{\rm{H}}}_{4}}^{+}+3{\rm{H}}{\rm{C}}{\rm{l}}{\rm{O}}\to {{\rm{N}}}_{2}+3{{\rm{H}}}_{2}{\rm{O}}+5{{\rm{H}}}^{+}+3{\rm{C}}{{\rm{l}}}^{-}$$

The removal efficiencies of color degree were higher than COD and NH_4_^+^-N (78–94%). The NaClO oxidation treatment (94%) was more effective than Fe^2+^ coagulation treatment (78%). Additionally, the removal efficiency of color degree by the NaClO enhanced Fe^2+^ coagulation treatment (92%) was similar the NaClO oxidation treatment, indicating that the organic matters with chromophore were mainly oxidized by NaClO.

**Figure 3:**
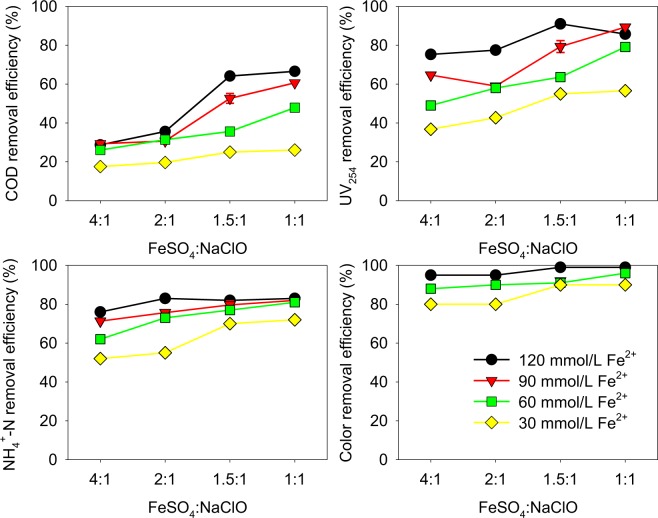
Removal efficiencies of COD, UV_254_, NH_4_^+^-N and color degree by NaClO enhanced Fe^2+^ coagulation with different dosages of Fe^2+^ and NaClO (Fe^2+^: 90 mmol/L, NaClO: 60 mmol/L, final pH = 8.3).

To obtain the optimum condition for the removal of COD, UV_254_, NH_4_^+^-N and color degree by NaClO enhanced Fe^2+^ coagulation treatment, different dosages of Fe^2+^ and NaClO were conducted (Fig. 3). It could be noticed that with the same dosage of Fe^2+^, the removal efficiencies increased with the increase of NaClO dosage. Generally speaking, when the mole ratios of Fe^2+^ to NaClO [M (FeSO_4_:NaClO)] were lower than 1.5:1, the removal efficiencies increased slowly. Thus, the optimum M (FeSO_4_:NaClO) was 1.5:1. With the same M (FeSO_4_:NaClO), the removal efficiencies increased with the increase of Fe^2+^ dosage, and did not significantly increase from 90 mmol/L to 120 mmol/L, especially for NH_4_^+^-N. Additionally, the sediment yield increased much with the increase dosage of Fe^2+^ dosage, probably due to the increase of the polymer yielded by Fe^2+^. Therefore, the condition of M (FeSO_4_:NaClO) = 1.5:1 and the dosage of Fe^2+^  = 90 mmol/L was deemed as the optimum. The removal efficiency of COD was 53%, the concentration of which decreased from 4700 mg/L to 2213 mg/L. The removal efficiency of UV_254_ was 79%, higher than that of COD, indicating that NaClO enhanced Fe^2+^ coagulation treatment was more effective for the removal of unsaturated organic matters determined by UV_254_. The removal efficiencies of NH_4_^+^-N was 80% with a final concentration of 93 mg/L. The removal efficiency of color degree was 92%.

### Further treatment with the Fenton oxidation and the photoelectro oxidation

To further remove the pollutants in the LLMC after the NaClO enhanced Fe^2+^ coagulation treatment, the Fenton oxidation and the PEO treatment was used. The supernatant of the effluent from the NaClO enhanced Fe^2+^ coagulation treatment was used for the further treatments.

#### Fenton oxidation treatment with different dosage of Fe^2+^ and H_2_O_2_

**Figure 4:**
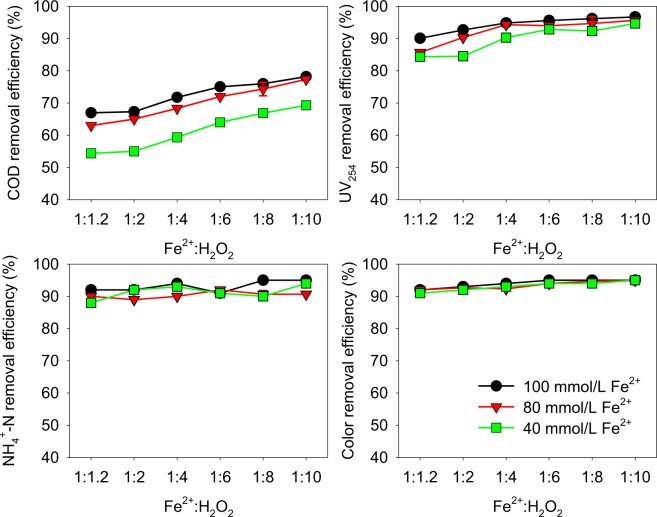
Removal efficiencies of COD, UV_254_, NH_4_^+^-N and color degree by Fenton oxidation with different dosages of Fe^2+^ and H_2_O_2_.

It is shown in Fig. 4 that the removal efficiencies of COD and UV_254_ increase with the increase dosage of Fe^2+^ and H_2_O_2_. More ·OH was generated with a higher dosage of Fe^2+^ and H_2_O_2_ Eqs (7 and 8)^22,23^, resulting to the higher removal efficiencies of the dissolved organic matters. However, as the concentrations of Fe^2+^ growing higher, the removal efficiencies increased slowly, because the ·OH was consumed by the surplus Fe^2+^ Eqs (9 and 10)^24^.

7 $${\rm{F}}{{\rm{e}}}^{2+}+{{\rm{H}}}_{2}{{\rm{O}}}_{2}\to {\rm{F}}{{\rm{e}}}^{3+}+{\rm{O}}{{\rm{H}}}^{-}+\cdot \,{\rm{O}}{\rm{H}}$$

8 $${\rm{O}}{\rm{r}}{\rm{g}}{\rm{a}}{\rm{n}}{\rm{i}}{\rm{c}}\,{\rm{P}}{\rm{o}}{\rm{l}}{\rm{l}}{\rm{u}}{\rm{t}}{\rm{a}}{\rm{n}}{\rm{t}}{\rm{s}}+\cdot \,{\rm{O}}{\rm{H}}\to {\rm{P}}{\rm{r}}{\rm{o}}{\rm{d}}{\rm{u}}{\rm{c}}{\rm{t}}{\rm{s}}$$

9 $${{\rm{H}}}_{2}{{\rm{O}}}_{2}+\cdot {\rm{O}}{\rm{H}}\to {\rm{H}}{\rm{O}}{\rm{O}}\,\cdot +{{\rm{H}}}_{2}{\rm{O}}$$

10 $${\rm{F}}{{\rm{e}}}^{3+}+{\rm{H}}{\rm{O}}{\rm{O}}\,\cdot \to {\rm{F}}{{\rm{e}}}^{2+}+{{\rm{O}}}_{2}+{{\rm{H}}}^{+}$$

It could be noticed that the dosage of Fe^2+^ of 100 mmol/L did not increase much the removal efficiency of the COD and UV_254_ (78% and 97%) compared with 80 mmol/L Fe^2+^ (77% and 96%), although the removal efficiencies of COD and UV_254_ in different conditions were significantly different (Paired sample t-test, p < 0.05) (Table S1). A higher dosage of Fe^2+^ would yield a higher amount of sludge. Therefore, the dosage of Fe^2+^ of 80 mmol/L was preferred. The highest removal efficiencies of COD and UV_254_ were obtained at the mole ratio of Fe^2+^ to H_2_O_2_ of 1:10. As a result, the best condition was the dosage of Fe^2+^ of 80 mmol/L and the mole ratio of Fe^2+^ to H_2_O_2_ of 1:10, from the aspect of COD (77%) and UV_254_ (96%). Compared with the Fenton oxidation (60%), the combined treatment of coagulation and Fenton oxidation for the removal of COD was much better (77%)^8^. The removal efficiencies of NH_4_^+^-N and color degree did not vary much with different dosage of Fe^2+^ and H_2_O_2_ (Paired sample t-test, p > 0.05) (Table S1). The final removal efficiencies of NH_4_^+^-N and color degree was 91% and 95%, respectively. Compared with the same treatment to the EMBR in our previous study, the final removal efficiency of COD was 86%, higher than the LLMC (77%)^12^. Thus, the PEO treatment was used in the following section.

#### Photoelectro oxidation treatment

**Figure 5:**
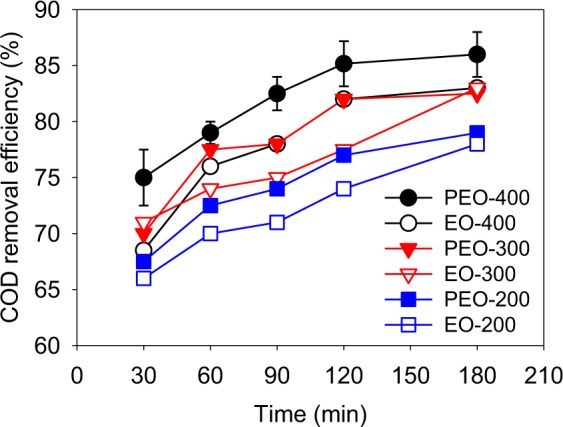
Removal of COD by photoelectro oxidation (PEO) and electro oxidation (EO) under different current density (200 A/m^2^, 300 A/m^2^, 400 A/m^2^) (PEO – 400 A/m^2^, final pH = 8.2).

In order to improve the removal efficiency of COD, the PEO was used for the treatment of LLMC after NaClO enhanced Fe^2+^ coagulation treatment. The EO was compared with the PEO treatment (Fig. 5). It could be noticed that under the same current density, the removal efficiencies of COD were higher in the PEO treatment than in the EO treatment. The removal efficiencies of COD increased with the increase current density from 200 A/m^2^ to 400 A/m^2^, both in the PEO and EO treatment. After 180 min, the final removal efficiency of COD was 86% with a final concentration of 660 mg/L. The generation of active chlorine in the PEO was higher than that in the EO treatment (Fig. S1), which might result to the higher removal efficiency of COD in the PEO treatment.

**Figure 6:**
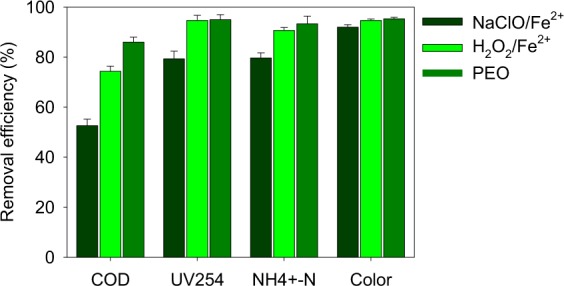
Final removal efficiencies of COD, UV_254_, NH_4_^+^-N and color degree by NaClO enhanced Fe^2+^ coagulation (NaClO/Fe^2+^), NaClO/Fe^2+^ combined with Fenton oxidation (H_2_O_2_/Fe^2+^) and NaClO/Fe^2+^ combined with photoelectro oxidation (PEO) treatment.

Compared with the Fenton oxidation treatment after the enhanced coagulation treatment, the removal efficiencies of COD were about 10% higher in the PEO treatment (Fig. 6). While the removal efficiencies of UV_254_, NH_4_^+^-N and color degree were relatively similar between the two treatments. Compared with ozonation (56% for COD and 0% for NH_4_^+^-N) and Fenton oxidation (60% for COD), the combination of PEO and enhanced coagulation treatment were more effective for the removal of COD and NH_4_^+^-N in the LLMC (86% and 93% respectively)^5,8,17^.

The reusability performance of the electrode was tested. The removal efficiencies of COD, UV_254_, NH_4_^+^-N and color degree did not vary much (sd < 3%) for the PEO treatment at 400 A/m^2^ (Figs 6 and S2). The RuO_2_/Ti mesh anode was a kind of dimensionally stable anode (DSA) usually used for electrolytic industry. The electrical conductivity, catalytical activity and stability were rather satisfied.

#### The generated halogenated intermediates in the photoelectro oxidation treatment

**Figure 7:**
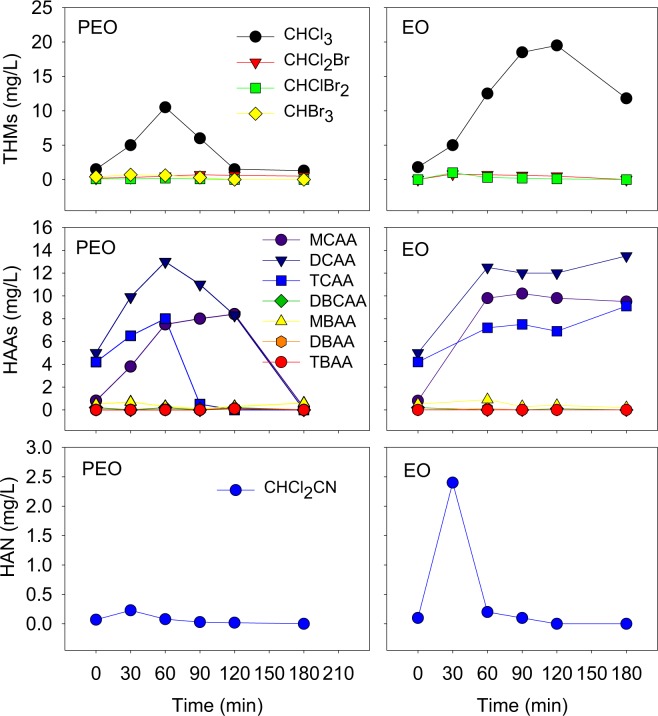
Concentrations of halogenated intermediates in the PEO and EO treatment.

The halogenated intermediates could be formed from the reaction of DOM with halogen. The halogenated intermediates in drinking water were largely investigated, but in the landfill leachate were seldom concerned. Due to the large amount of active chlorine generated in the PEO and EO process and the high concentration of COD in the LLMC, the major kinds of intermediates, including the trihalomethanes (THMs: CHCl_3_, CHCl_2_Br, CHClBr_2_ and CHBr_3_), haloacetic acids (HAAs: C_2_H_3_O_2_Cl-MCAA, CH_2_O_2_Cl_2_-DCAA, C_2_HO_2_Cl_3_-TCAA, CH_2_O_2_BrCl-DBCAA, C_2_H_2_O_2_Br-MBAA, CH_2_O_2_Br_2_-DBAA and C_2_HO_2_Br_3_-TBAA) and haloacetonitriles (HANs: CHCl_2_CN) were measured in the PEO and EO process (Fig. 7).

Generally speaking, both in the PEO and EO treatment, the concentrations of the formed intermediates with more chlorine were higher than those with more bromine, due to the generation of the active chlorine. The result was similar to a previous study^25^. In the PEO treatment, regarding the THMs, the concentration of CHCl_3_ increased to 10.5 mg/L in 60 min and decreased nearly to 0 mg/L in 120 min. As to the HAAs, the concentrations of MCAA, DCAA and TCAA increased to 8.0–13.0 mg/L in the first 60–120 min and decreased nearly to 0 mg/L in 180 min. Similar to the THMs, almost no HAAs with bromine was formed during the process. For HAN, only CHCl_2_CN was detectable. The highest concentration achieved in the first 30 min (0.2 mg/L), was lower than those of THMs and HAAs without bromine. In 180 min, the concentration of CHCl_2_CN decreased to 0 mg/L. The formation and degradation trends of the halogenated intermediates were different in the EO process. Regarding the THMs, the concentrations of CHCl_3_ increased to 19.5 mg/L in 120 min, and decreased to 11.8 mg/L in 180 min. As to the HAAs, the concentrations of MCAA, DCAA and TCAA increased to 7.2–12.5 mg/L in the first 60 min, and almost keep steady to 180 min. The concentration of CHCl_2_CN (HAN) was 2.4 mg/L at 30 min, and decreased to 0 mg/L in 180 min. Therefore, intermediates with chlorine and without bromine could be formed during the PEO and EO treatment. The formed chlorinated intermediates could be eliminated in 180 min in the PEO treatment. However, most of the formed chlorinated intermediates still existed in 180 min in the EO treatment, indicating that the PEO treatment was more effective for the elimination of the chlorinated intermediates than the EO treatment.

It should also be noticed that before the treatment of the PEO and EO, some halogenated compounds existed with concentrations ranging from 0.1 to 5.0 mg/L, indicating that the some halogenated compounds was formed in the NaClO enhanced Fe^2+^ coagulation treatment. After the PEO treatment, the halogenated intermediates formed both in the NaClO enhanced Fe^2+^ coagulation and PEO treatment were eliminated. Therefore, from the aspect of the halogenated intermediates, the NaClO enhanced Fe^2+^ coagulation treatment combined with the PEO treatment was suitable for the treatment of the LLMCs.

In conclusion, the most suitable technique for treatment of the LLMC was NaClO (60 mmol/L) enhanced Fe^2+^ (90 mmol/L) coagulation followed with the PEO treatment (RuO_2_/Ti mesh anode and Ti mesh cathode) for 3 hours at a current density of 400 A/m^2^. The final removal efficiencies of COD, UV_254_, NH_4_^+^-N and color degree in the LLMC were 86%, 95%, 93% and 95%. All the formed halogenated compounds were eliminated.

## Materials and Methods

### Materials

**Figure 8:**
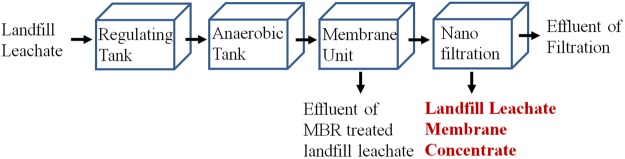
Treatment process of the landfill leachate.

The LLMC and EMBR samples were collected from a sanitary landfill in Chaoyang district in Beijing. The landfill was built in 2002 and disposed 3400 tons of municipal solid waste per day. The landfill leachate was treated by two-state upflow anaerobic sludge blanket and the MBR followed with nanofiltration membrane (Fig. 8). The EMBR was collected for characteristic comparison with the LLMC. All the samples were stored at 4 °C. The samples were analyzed and used for treatment in 10 days. The initial characteristics of these samples are listed in Table 1. All the samples were measured in triplicates, with a standard deviation of less than 3%.

### Experimental procedure

The LLMC of 300 mL was used for each NaClO enhanced Fe^2+^ coagulation treatment. The given amounts of NaClO and FeSO_4_·7H_2_O were added into the LLMC simultaneously. The mixture was fast stirred for 2 min (200 r/min), and then slowly stirred for 20 min (40 r/min). After the experiment finished, the mixture was settled for 60 min. Then, the supernatant was filtered by 0.45 μm membranes for analysis. The effluent from Fe^2+^/NaClO treatment (100 mL) was treated by Fenton oxidation. The given amounts of H_2_O_2_ and FeSO_4_·7H_2_O were added into the effluent. The mixture was stirred for 60 min. Similarly, the supernatant was passed through 0.45 μm filters before analysis. The effluent from Fe^2+^/NaClO treatment (100 mL) was also treated by the PEO treatment. The RuO_2_/Ti mesh (60 m^2^) was used as the anode, and Ti mesh (60 m^2^) was the cathode. The PEO device was similar to our previous study^26^. The device was briefly described here. The distance between the 2 electrodes was 5.0 cm. A UV lamp (9 W, low-pressure mercury, wavelength 254 nm) in a quartz glass tube was placed in the center of the 2 electrodes. The current density was set at 200 A/m^2^, 300 A/m^2^ and 400 A/m^2^. These experiments were conducted in 180 min.

### Analytical method

The basic water quality index was measured based on the water and wastewater detection and analysis methods^27^. COD was measured by heating digestion colorimetry method using a HACH DRB200 coupled with a DR2800 attachment. UV_254_ was measured by an ultraviolet spectrophotometry. NH_4_^+^-N was tested using the sodium reagent method. Color degree was the value of absorbance at λ_max_ = 436 nm measured with an ultraviolet spectrophotometry. Cl^−^ was measured using ion chromatography. Active chlorine was measured using the N, N- two ethyl benzene two amines spectrophotometric method.

The dissolved organic matter (DOM) was separated based on a previous method^28^. After the samples filtered with 0.45 μm membranes, XAD-8 resin was used for the separation of the hydrophobic bases (HOB), hydrophobic acids (HOA), hydrophobic neutrals (HON) and hydrophilic matter (HiM).

The halogenated intermediates were extracted using hexane for the trihalomethanes (THMs), and using methyl tert-butyl ether for the haloacetic acids (HAAs) and haloacetonitriles (HANs). All these compounds were measured using a GC-ECD. A HP-5 fused silica capillary column (30 m × 0.32 mm × 0.25 μm) was used. Samples (1 μL) were injected in splitless mode. The carrier gas was high purity nitrogen. For the detection of THMs, the velocity of nitrogen was 6.3 mL/min. The injector and detector temperatures were 200 °C and 290 °C, respectively. The initial temperature was set at 35 °C (held for 4 min) and to 260 °C at a rate of 10 °C/min (held for 4 min). For the detection of the HAAs and HANs, the velocity of nitrogen was 2.0 mL/min. The injector and detector temperatures were both set as 250 °C. The temperature program procedure was: 35 °C (held for 4 min) to 65 °C at a rate of 2 °C/min.

## Electronic supplementary material

Supplementary information
