# Supplementary material for: Characterization and treatment of landfill leachate membrane concentrate by Fe2+/NaClO combined with advanced oxidation processes
Source: Sci Rep. 2018 Aug 21;8:12525. doi: 10.1038/s41598-018-30917-5 (PMC6104102; doi:10.1038/s41598-018-30917-5)
Supplement: Supplementary file 1 — Supplementary information [file 41598_2018_30917_MOESM1_ESM.docx]

**Supporting information**

**Characterization and treatment of landfill leachate** **membrane concentrate by Fe^2+^/NaClO combined with advanced oxidation processes**

Meng Qiao, Xu Zhao*, Xiaoyun Wei

*Key Laboratory of Drinking Water Science and Technology, Research Center for Eco-Environmental Sciences, Chinese Academy of Sciences, Beijing 100085, China*

^*^ Corresponding author:

Xu Zhao; Tel.:+86 10 62849667; E-mail address: [zhaoxu@rcees.ac.cn](mailto:zhaoxu@rcees.ac.cn).

Table S1 Paired t-test for the removal efficiencies by Fenton oxidation

| P | 100 VS 80 mmol/L Fe^2+^ | 80 VS 40 mmol/L Fe^2+^ |
| --- | --- | --- |
| COD | 0.003** | 0.000** |
| UV_254_ | 0.012* | 0.010* |
| NH_4_^+^-H | 0.021* | 0.434 |
| Color | 0.175 | 0.076 |

**: P < 0.01, *: P < 0.05

Figure S1 Concentrations of Cl- formed both in the PEO and EO processes at a current density of 400 A/m^2^

Figure S2 Removal efficiency of COD using the same electrodes by PEO treatment at 400 A/m^2^ for three times
